# Preliminary feasibility torque mechanical evaluation for 3D printed orthodontic springs with different parameters: in vitro study

**DOI:** 10.1186/s12903-021-01473-8

**Published:** 2021-03-07

**Authors:** Ahmed Othman, Steven Hartman, Dragan Ströbele, Jassin Arnold, von See

**Affiliations:** Digital Technologies in Dentistry and CAD/CAM Department, Danube Private University, Steiner Landstraße 124, 3500 Krems an der Donau, Austria

**Keywords:** CAD/CAM, 3D printing, Digital orthodontics, Torque, Springs

## Abstract

**Background:**

The purpose of the presented investigation is to evaluate the resulting torque on loaded 3D printed springs using different coil thickness and length.

**Methods:**

Specimens were designed and printed using the 3D printer MAX (Asiga, Sydney, Australia) with 3D printable, experimental, flexible material (Code:BM2008, GC, Tokyo, Japan). The specimens were divided into three groups according to spring coil design. Control group (n = 18), length group (n = 19) and thickness group (n = 22). Groups were tested using a Sauter Machine for torque calculation (DB, Grindelwald, Switzerland) in conjunction with a universal testing machine (Zwick Z010, Ulm, Germany) for clock-wise and anti-clockwise testing. Statistical analysis was performed using the Steel–Dwass test to compare median values of the three groups in both testing directions (p < 0.001).

**Results:**

The highest torque value was determined in the thickness group for both clockwise and anti-clockwise testing directions, achieving 44.00 N/mm and 39.62 N/mm respectively. The length group ranged from 21.65 to 11.04 N/mm in clockwise direction and from 18.04 to 11.38 N/mm in counter-clockwise testing. The control group ranged from 22.72 to 17.18 N/mm in the clock-wise direction while in the anti-clock wise testing it ranged from 21.34 to 16.02 N/mm.

**Conclusions:**

The amount of torque produced from the computer aided designing/computer aided manufacturing (CAD/CAM) springs is being affected by diameter more than the length design parameter in comparison to the control group. The values of the thickness group are significantly higher than those of the length group (P < 0.001).

## Background

The advances in dentistry and malocclusion corrections for development of a healthy occlusion have led to an increased demand of orthodontic treatment. Malocclusion can vary depending on certain factors including the dental angle classification, skeletal classification and genetic factors [1–3]. Orthodontic movements vary depending on the degree of malocclusion, other biological factors such as age and suspected treatment planning [2–5].

The usage of different orthodontic spring designs as the T or delta loops made of titanium-molybdenum or super elastic nickel titanium material could be appropriate for producing a clinically usable forces which could provide mechanical teeth retraction [6–8]. The usage of CAD/CAM in orthodontics is nowadays not only combined with aligner virtual production but also appliance design and manufacturing for fixed and removable functional devices [9, 10]. Brackets and wires now can be fabricated by CAD/CAM technology on a virtual setup designed before commencing with the treatment. Thus, the final product is a precise duplicate of pre-treatment digital setup greatly diminishing chances for error [11]. Digital orthodontic treatment is being presented in literature for clinical usage as CAD/CAM technology is nowadays being directly involved in dentistry [12–14]. Digital fabrication of fixed functional orthodontic appliances is clinically proven for its successful results [15]. However, digital designing and printing of orthodontic springs has until now not been presented in literature nor tested. Accordingly, having the possibility of producing and providing such material for individualized orthodontic usages will increase with further testing and thus might be considered. The necessary requirements are influenced by the individual design of each spring and the ability to attach and support the spring to the tooth surface.

The 3D printing resin material for orthodontic springs is a recent development with neither data nor studies available for its efficiency.

Accordingly, the aim of the present investigation was to mechanically evaluate the torque of the digitally designed and 3D printed orthodontic springs in vitro by having differently designed orthodontic spring groups prior to clinical application.

## Methods

In the present investigation, spring specimens were 3D printed using MAX (Asiga, Sydney, Australia) with DLP (digital light processing) technology via the experimental flexible printable material (Code:BM2008, GC, Tokyo, Japan) which contains a 3D resin material as its main ingredient. The specimens were designed by the computer aided program Autodesk Netfabb (San Rafael, CA, USA). The post processing of the specimens was performed by GC-Europe according to the manufacturer’s instructions. Unheated ultrasonic bath (Bandelin, Berlin, Germany) reusable isopropanol solution with a concentration of 96% was utilized to clean the specimens for 2 min followed by 2 more minutes of a clean isopropanol bath with the same concentration.

The specimens were withdrawn from the solution and dried with compressed air in-between the two cleaning cycles. Surface polymerization was done using Labolight DUO (GC, Tokyo, Japan) with double wavelength LED technology in a range of 380–510 nm with spectrum range peaks of 465–485 nm (12 Blue LED’s) and 390–400 nm (3 Violet LED’s). Two periods of 3-min durations each were applied, and the samples were turned for curing from both sides. After post curing, carbide bur and nipper were used to remove supports.

**Fig. 1:**
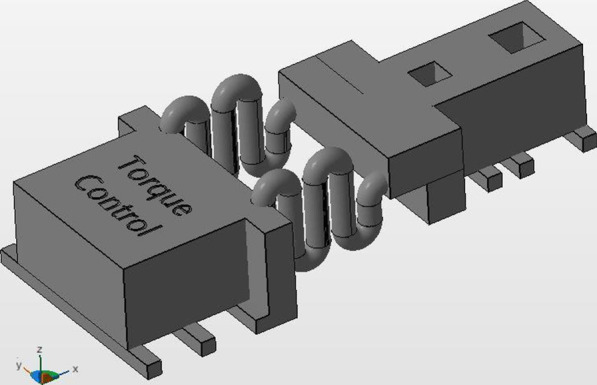
Control group

**Fig. 2:**
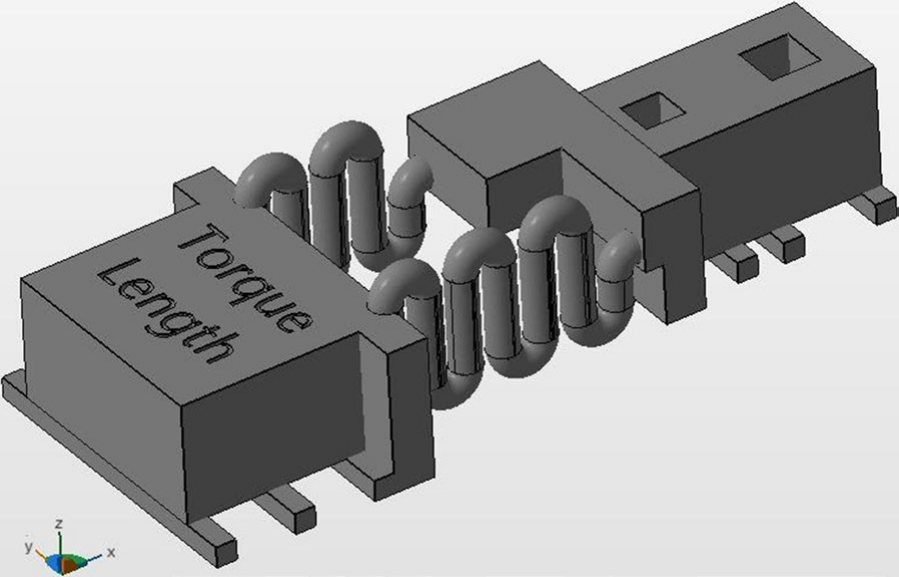
Length group

**Fig. 3:**
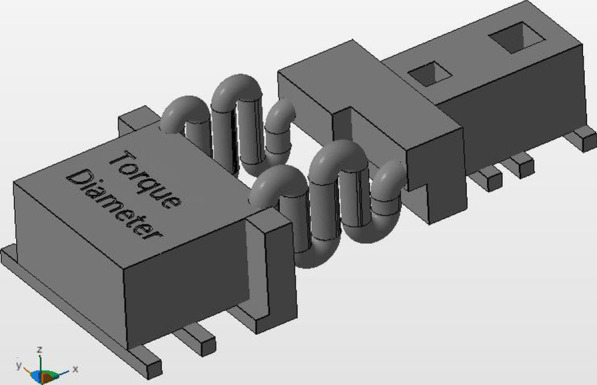
Diameter group

After eliminating all specimens’ printable supports, the specimens were divided into three different groups according to their different design parameters, control group (n = 18), length group (n = 19) and thickness group (n = 22) (Table 1; Figs. 1, 2, 3). The specimen groups did not have equal numbers due to springs destruction during the post-processing.

**Table 1:** Overview of the group classification

Group	Control group	Length group	Diameter group
Specimens	18 specimens	19 specimens	22 specimens
feature	Two identical springs	Different coil numbers (n1 = 4, n2 = 6)	Different coil radius (Δρ = 0.15 mm)

The control group consisted of the same material and design parameters used in recent study conducted by Othman et al. who found that the 3D resin used material provides an acceptable force in comparison to NiTi material [16]. Accordingly, the control group was characterized by two in design identical springs with radius 0.75 and four coils, while the length group was characterized by two springs with a difference in coil-number (n_1_ = 4, n_2_ = 6) and the diameter group was defined by two coils with different radius (Δr = 0.15 mm).

**Fig. 4:**
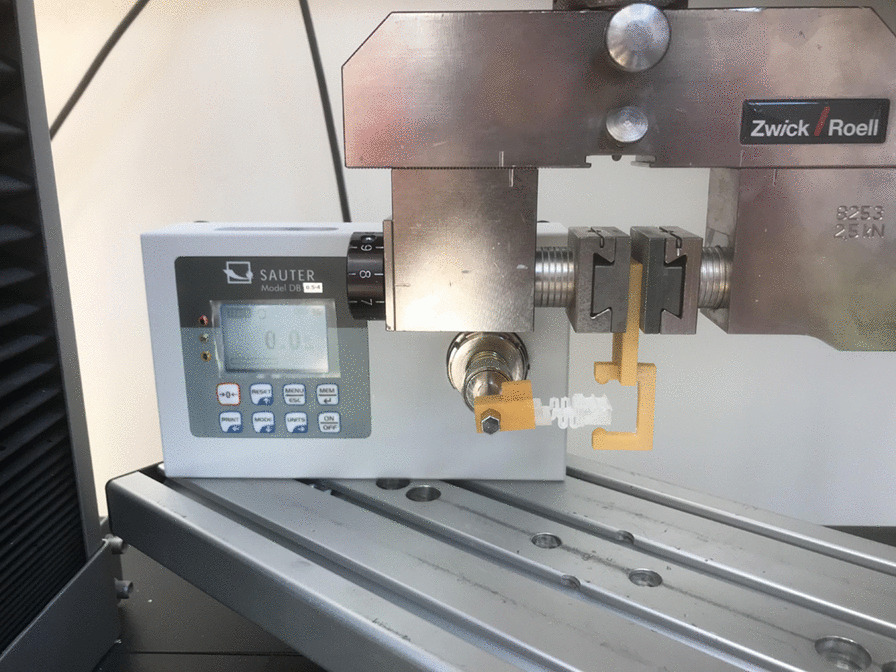
Testing process (Seen above, the C shaped construct and the reference key locking a spring in place for testing with the Z010 Zwick/Roell (Ulm, Germany) upon initiation the C shaped will be shifted up and down so that each end delivers forces onto the spring which are then recorded by torque device (Sauter DB 0.5-4, Grindelwald, Switzerland) this process is repeated five times total in both directions for every spring tested)

A reference key was designed using Autodesk Netfabb (San Rafael, CA, USA) and printed with the Varseowax Model material using Varseo S 3D printer (BEGO, Bremen, Germany), which acted as a spring’s attachment to the torque measuring device (Sauter DB 0.5-4, Grindelwald, Switzerland) in which the values were automatically recorded with an implemented software in the device itself. In order to assure pointed force exertion upon the springs base, a C shaped 3D printed key was loaded into the universal testing machine (Z010 Zwick/Roell, Ulm, Germany). The C shaped key was designed using Autodesk Netfabb (San Rafael, CA, USA) and printed with the Varseowax Model material using Varseo S 3D printer (BEGO, Bremen, Germany) (Fig. 4).

Parameters for the universal testing machine were predefined by speed starting position with 50 mm/min and 5 s holding time for torque measuring between the clockwise and counter clock wise directions. In the torque measuring device, the reference key which acted as a holder for the specimens was attached to the device and loaded in the universal testing machine for measuring both directions for five cycles each (Fig. 4). All results of both clockwise and counter clockwise force exertion was recorded in N (Newton) using the torque measuring device itself which has an internal software for displaying the values and the maximum for each specimen were recorded.

### Descriptive analysis

Results were divided in the three groups: control, length and thickness with clockwise as well as counter clockwise testing for each of the three groups. Statistical analysis was performed via Steel–Dwass test which is primary used to evaluate non-parametric distributions of multiple comparisons to determine which ones are different. The non-parametric Steel–Dwass statistical method was utilized for all groups in both testing directions, clock and counter clockwise, by calculating the median. Accordingly, in this study the median values of the three groups in both testing direction were compared (p < 0.001).

## Results

The compiled data that was gathered from the measurements was to be compared and analyzed for statistical analysis between the groups. The values of both clockwise and counter clock-wise data sets were calculated separately (Tables 2, 3).

**Table 2:** clockwise torque values

Groups	Control	Length	Diameter
Minimum torque	17.1	11.1	25.2
Maximum torque	23.5	21.8	44.2
Mean	19.79	16.65	32.55
Significance to the control group		p < 0.001	p < 0.001

**Table 3:** Counter clockwise torque values

Groups	Control	Length	Diameter
Minimum torque	15.2	10.7	20.2
Maximum torque	21.8	18.3	40.3
Mean	17.72	14.11	26.18
Significance to the control group		p < 0.001	p < 0.001

The statistical analysis showed significant difference between the three groups of springs (p < 0.001). Clockwise and counter-clock wise mechanical testing both proved significant differences for the diameter group (p < 0.001). The highest measured values found for the diameter group was 44.00 N/mm. The lowest scores noted was within the length group 11.04 N/mm.

The clock and counter clockwise for the diameter group showed a significant value when compared to both control and length groups using the steel–Dwass test (p < 0.001).

Finally, there are significant differences within each group between clock-wise and counter-clockwise directions (p < 0.05).

## Discussion

The aim of the present investigation was to mechanically evaluate the torque resulted from the digitally designed and 3D printed orthodontic springs with different design parameters by mechanical loading in clock-wise and counter-clockwise direction. It was proven, that the thickness group had a highly significant torque value in clock wise than in counter-clockwise direction.

The study protocol followed the manufacturer’s instructions for post processing, which ensured uniform results. Furthermore, torque testing was used to find out whether the designed springs can develop sufficient deflection processes to evaluate the tooth movement possibility with a 3D printed spring. Contrary to the current metallic springs, where a simple deflection process results in an acceptable force for orthodontic usage [6].

Printed springs may need a deflection processes to develop sufficient forces to enable tooth movement possibility with a 3D printed material, which could lead to a customized usage of the springs depending on the malocclusion problem and treatment aims and objectives. However even with increased effort, torque and force still aid orthodontic treatment [17, 18]. Clinical use cannot be simulated entirely in vitro with standardized tests, but it’s possible to find material-specific properties in vitro [19]. In the present investigation, significant difference between the groups for 3D printed springs was found.

This study is in line with Chudasama et al. [20] who determined that wire size has a direct effect on the amount of exerted force. Further studies and experiments should be undertaken to investigate this effect in depth.

A study conducted by Ubirajara et al. showed that spring parameters are greatly influencing the mechanical effect [6]. It is difficult to compare the effect of the printed springs to the laboratory constructed springs because of the material used and the construction method. This is due to the fact that every study is performed with different devices as well as with different operators. For this reason, the absolute data for torque and the obtained values can be compared only inside the same study. In a study conducted by Davidović et al. [21], closed coil springs made of nickel titanium material were compared to elastic chains. A similar approach for three dimensionally printed springs could be undertaken to determine their efficiency when paired against other materials on the orthodontic field. Another study, performed by Barwart et al. [22] tested the super elasticity of nickel titanium springs under different temperatures. Such a study would prove invaluable for understanding how the 3D printed material endures in vivo, as oral temperature fluctuations occur, for instance during meals [19]. A third study by Nattrass et al. [23] explored the environmental factors that influence both elastomeric chain and nickel titanium springs. The independent design parameter for orthodontic springs could be clinically relevant. Accordingly, this study should be an additive research in the recent digital orthodontics investigations and techniques improvement. As limitations of present study, the chemical polymerization process and artificial aging within the printable resin needs further investigation. Also, the amount of friction between the springs and reference key was not measured because of the inability to determine this amount of friction precisely.

This study is preliminary a feasibility testing of the 3D printed material in which the parameters should be evaluated in depth before any clinical consideration.

## Conclusion

The findings showed a higher torque value for the diameter group compared to the control group and a clear increase of value in clockwise direction for diameter group with mechanical testing. The length group did not show a significant difference between the two testing directions.

## Data Availability

The datasets used and/or analysed during the current study are available from the corresponding author on reasonable request.

## Abbreviations

3D: Three dimensionally
CAD/CAM: Computer aiding designing/computer aided manufacturing
DLP: Digital light processing
NiTi: Nickel titanium
